# Effect of semaglutide and empagliflozin on cognitive function and hippocampal phosphoproteomic in obese mice

**DOI:** 10.3389/fphar.2023.975830

**Published:** 2023-03-17

**Authors:** Xiaoyi Chen, Shuchun Chen, Zelin Li, Ruiyi Zhu, Zhuoya Jia, Jiangli Ban, Ruoxi Zhen, Xing Chen, Xiaoyu Pan, Qingjuan Ren, Lin Yue, Shu Niu

**Affiliations:** ^1^ Department of Internal Medicine, Hebei North University, Zhangjiakou, China; ^2^ Department of Endocrinology, Hebei General Hospital, Shijiazhuang, China; ^3^ Department of Internal Medicine, Hebei Medical University, Shijiazhuang, China

**Keywords:** semaglutide, empagliflozin, phosphoproteomic, obesity, cognitive impairment

## Abstract

**Objective:** Based on the 4D label-free phosphoproteomic technique, we examined the differences in cognitive function and hippocampal phosphorylated protein expression in high-fat diet-induced obese mice after the intervention of semaglutide and empagliflozin, as well as the effects of both on protein activity and function in obese mice’s hippocampal tissues and the signaling pathways involved.

**Methods:** Thirty-two C57BL/6JC male mice were assigned to two groups randomly: A control group (group C, 10% of energy is from fat, n = 8) and a high-fat diet group (group H, 60% of energy is from fat, n = 24). The high-fat diet-induced obese mice were screened after 12 weeks of feeding based on the criterion that the bodyweight of mice in fat rich diet group was greater than or equal to 20% of the average body weight of the mice in the blank control group. Group H separate into group H (n = 8), group Semaglutide (group S, n = 8), and group empagliflozin (group E, n = 8). For a total of 12 weeks, group S received 30 nmol/kg/d bodyweight of semaglutide intraperitoneally, group E received 10 mg/kg/d bodyweight of empagliflozin *via* gavage, and groups C and H received equal amounts of saline by intraperitoneal injection and gavage. At the end of treatment, the mice were appraised for cognitive function employing the Morris water maze (MWM), and serum fasting glucose, lipids, and inflammatory parameters were measured. The 4D label-free phosphoproteomics method was employed to screen the differential phosphoproteins and loci in hippocampal tissues of mice in different treatment groups, and bioinformatics was used to analyze the biological processes, signaling pathways, and related protein–protein interaction (PPI) network analysis of these differentially phosphorylated proteins.

**Results:** In comparison to normal controls, The escape latency of obese mice induced by high-fat diet was prolonged, the percentage of swimming time in the target quadrant was reduced, and the number of times of crossing the platform was reduced, whereas semaglutide and empagliflozin treatment reduced escape latency, increase the percentage of swim time in the target quadrant and increase the frequency of passing through the platform area, although there is little difference in the effect of the two drugs. The phosphoproteomic results showed 20,493 unique phosphorylated peptides, representing 21,239 phosphorylation sites and 4,290 phosphorylated proteins. Further analysis revealed that the proteins corresponding to these differentially phosphorylated sites are jointly distributed in signaling pathways such as dopaminergic synapses and axon guidance, and are involved in biological processes such as neuronal projection development, synaptic plasticity, and axonogenesis. Notably, the key factors voltage-dependent L-type calcium channel subunit alpha-1D (CACNA1D), voltage-dependent P/Q-type calcium channel subunit alpha-1A (CACNA1A), and voltage-dependent N-type calcium channel subunit alpha-1B (CACNA1B) were all found to be involved in the dopaminergic synapse pathway, and their expression was upregulated by semaglutide and empagliflozin.

**Conclusion:** We found for the first time that a high-fat diet decreased CACNA1D, CACNA1A, and CACNA1B protein serine phosphorylation, which may affect neuronal development, synaptic plasticity, and cognitive function in mice. Notably, semaglutide and empagliflozin increased the phosphorylation of these proteins.

## 1 Introduction

Obesity is associated with energy imbalance in the body, which is also a chronic metabolic disease. In 2016, there were over 670 million obese adults and around 1.9 billion overweight people globally. Between 2002 and 2012, the adult obesity rate in China increased from 29.9% to 42.0% (NCD Risk Factor Collaboration, 2017). The WHO has classified obesity as a disease, making it one of the biggest public health problems of the 21st century. Obesity is closely related to chronic diseases such as cancer, diabetes and cardiovascular diseases, and is as well as a risk factor for numerous chronic diseases. The recent proof indicates a linear relationship between obesity and cognitive deficits, and an increased risk of dementia and Alzheimer’s disease (AD) later in life as well (Solas et al., 2017). Studies in animal models of obesity have shown that SD rats exhibit impaired learning and cognitive function with decreased neuronal and myelin viability after high-fat dietary feeding (Niepoetter et al., 2021). Previous literature has reported that the development of obesity-related cognitive impairment may be associated with inflammation, insulin resistance, disruption of the blood-brain barrier, and abnormal endocrine function, but more precise mechanisms need to be researched (Solas et al., 2017; Niepoetter et al., 2021). Therefore, it is important to examine the relationship between obesity and cognitive deficits, to further elucidate the pathogenesis of obesity-related cognitive impairment, and develop therapy methods for early prevention, diagnosis, and treatment of cognitive impairment.

Protein phosphorylation is essential for eukaryotic signaling and regulates the entire spectrum of life activities, including the cell cycle, development and differentiation, metabolism, and apoptosis, which is the most prevalent post-translational modification of proteins in cells (Delom and Chevet, 2006). Many important life activities and disease occurrences are controlled not only by the relative abundance of proteins but also by post-translational protein modifications. Therefore, in addition to studying the ‘bare’ proteome encoded by the genome, there is a need for in-depth studies of post-translationally modified proteins and the proteome’s regulatory processes (Mertins et al., 2013). The rapid development of mass spectrometry and phosphorylated peptide enrichment technologies in recent years has enabled the identification of protein phosphorylation profiles on a large scale. The 4D label-free quantitative proteomics technology (4D-LFQ) is a new generation of proteomics analysis based on protein isolation, mass spectrometry, and bioinformatics techniques. It allows for a comprehensive perspective of protein phosphorylation modifications in cells or tissues (He et al., 2019).

In addition to glucose regulation, glucagon-like peptide-1 (GLP-1) is a essential entero-insulin in the body with glucose-concentration-dependent pro-insulin secretory properties and plays a significant role in synaptic plasticity and memory formation (Ferreira, 2021). Semaglutide is a new generation of synthetic GLP-1 analog, which is used to remedy type 2 diabetes mellitus (T2DM) (Dhillon, 2018). Studies have found that semaglutide can decrease apoptosis and improve cognitive dysfunction in cerebrovascular disease (Yang et al., 2019), Parkinson’s disease (PD) (Zhang et al., 2019), and AD (Chang et al., 2020). However, the effect of semaglutide on obesity-related cognitive function has not been reported. Empagliflozin, a sodium-glucose co-transporter 2 (SGLT-2) inhibitor, has been shown to ameliorate cognitive function in mice with AD and diabetes, moreover, it can effectively lowering glucose and improving cardiovascular outcomes. The potential mechanism for the beneficial effect of empagliflozin on cognition are bound up with reduced oxidative stress in the brain and inhibition of neurological inflammation in these models (Lin et al., 2014; Hierro-Bujalance et al., 2020). However, to the best of our knowledge, no studies have been published on the effects of empagliflozin on hippocampal synaptic plasticity and cognitive function in high-fat dietary feeding obese mice. Furthermore, the effects of semaglutide and empagliflozin on hippocampus phosphorylation proteome and mechanisms of action in high-fat dietary feeding obesity have never been studied.

This study was the first to observe a difference in hippocampal phosphorylated protein expression in high-fat diet-induced obese mice before and after the intervention of semaglutide and empagliflozin using the phosphorylated 4D-LFQ technique, allowing researchers to further investigate the effects of both on hippocampal cognitive function in obese mice and provide a new basis for the precise prevention and treatment of obesity-related cognitive disorders.

## 2 Materials and methods

### 2.1 Ethics statement

This study was conducted in accordance with the NIH guidelines for the care of laboratory animals and the related guidelines, and it received approval by the Animal Ethics Committee of the Hebei General Hospital (No. 202173).

### 2.2 Animal model

Male C57BL/6JC mice (age: 6 weeks) were purchased from Hebei *In vivo* Biotechnology Co., Ltd [License No: SYXK (Jun) 2015-0004] and housed in the Experimental Animal Centre of the Hebei General Hospital, with four mice in each cage, incubated at 22°C ± 2°C, 55% ± 10% humidity, and a light/dark cycle of 12:12 h. Food and water were made freely available throughout the experimental period.

After 1 week of acclimatization, the animals were categorized into a blank control feeding group (group C, n = 8) and a high-fat dietary feeding group (group H, n = 24) by using the random number table method. The control group was fed a diet consisting of 20% protein, 70% carbohydrate, and 10% fat, while the high-fat diet group was fed a diet consisting of 20% protein, 20% carbohydrate, and 60% fat. The mice in each group received equal numbers of calories daily and drank water *ad libitum*. After 12 weeks of high-fat feeding, the mice in the a high-fat dietary feeding group were screened for high-fat diet-induced obesity considering that their bodyweight was ≥20% of the mean body weight of mice in the control group. Group H was further categorized into group H (n = 8), group H + semaglutide (group S, n = 8), and group H + empagliflozin (group E, n = 8). Then, 30 nmol/kg/day bodyweight of semaglutide was administered intraperitoneally to group S and 10 mg/kg/day bodyweight of empagliflozin was administered *via* gavage to group E. Then, equal amounts of saline were administered intraperitoneally to the mice in groups C and H for 12 weeks in total. After completion of the drug intervention, the mice fasted for 12 h, and blood was withdrawn from the tail vein to determine the fasting glucose level.

### 2.3 Morris water maze (MWM) test

After completing the intervention, all mice were tested by MWM (Shanghai Jiliang Software Science & Technology Co., Ltd, Shanghai, China) to assess their cognitive function status, such as learning and memory. The device consisted of a white circular pool of diameter 120 cm and a depth of 45 cm. A pool filled with water at a temperature of 23°C ± 1°C, and a platform installed 1–2 cm under the water surface. The test consisted of two phases: Four consecutive days of positioning navigation training and a fifth day of space exploration training. In the first phase, the mice were randomly placed into the water from one of the four quadrants each day to search for an underwater escape platform, and the time taken by the mice to swim to and find the platform served as the escape latency period. Then, the platform was removed for spatial exploration training. The mice were placed diagonally from the original platform into the water and they searched the platform for 60 s. Their swimming trajectory, the number of times they passed through the original platform, the percentage of time they lingered the target quadrant, the total distance and the average swimming speed were recorded and counted within 60 s.

### 2.4 Sample collection and preparation

Blood was collected from the eyes of the mice after overnight fasting and then centrifuged at 3,000 rpm for 10 min at 4°C so as to collect the serum and store it at −80°C. After blood collection, the mice were decapitated and executed, the bilateral hippocampal regions were quickly separated on an ice tray, and the surface water was blotted out and frozen at −80°C.

### 2.5 Serum indicator testing

The levels of interleukin-6 (IL-6) and IL-1β (IL-1β) in mice were measured by antibody sandwich ELISA (Multi Sciences Biotech Co., Ltd., Hangzhou, China); the concentration of total cholesterol (TC), triglyceride (TG), fasting plasma high-density lipoprotein (HDL-C), and fasting plasma lipoprotein (HDL-C) in the mice were measured by enzymatic method using commercially available kits (Nanjing Jian Cheng Bioengineering Institute, Nanjing, China).

### 2.6 Protein extraction and standardization

The hippocampal tissues from the mice were well ground with liquid nitrogen, removed into a 1.5-mL centrifuge tube, made to a final concentration of 1 mM with a protease inhibitor (Biotech, A610425-0005) and phosphatase inhibitor (Roche, 4906837001), followed by lysing by sonication (SCIENTZ-II D). The solution was then centrifuged at ×12,000 g for 10 min at room temperature to remove the cell debris, and the supernatant was removed and then centrifuged again to remove the supernatant. Next, the supernatant was transferred into a new centrifuge tube and the BCA kit was applied for protein concentration determination. Then, 10 μg of the protein was removed from each sample and segregated by 12% SDS-PAGE. The segregated gels were stained with Komas Brilliant Blue using the eStain LG Protein Stainer (Nanjing Kingsray Biotechnology Co., Ltd., Nanjing, China), and the stained gels were imaged using a fully automated Digital Gel Image Analysis System (Shanghai Tennant Technology Co., Ltd., Shanghai, China).

### 2.7 Protein digestion and peptide labeling

Each sample was diluted to the same concentration and volume with a lysis solution. Dithiothreitol (DTT) was added to the abovementioned protein solution to a final concentration of 5 mM and then reduced at 56°C for 30 min. Next, a corresponding volume of iodoacetamide was added to a final concentration of 10 mM and left for 15 min at room temperature away from light. To this solution, 6 times the volume of acetone was added to precipitate the protein and then left overnight at −20°C. The precipitate was collected *via* centrifugation at ×8,000 g for 10 min at room temperature, and acetone was evaporated for 2–3 min, followed by re-solubilization of the precipitate with ammonium bicarbonate (50 mM), the addition of trypsin in a 50:1 mass ratio (protein: trypsin), enzymatic digestion overnight at 37°C, and collection by vacuum centrifugation, to be followed by desalting and lyophilization.

### 2.8 Enrichment of phosphorylated peptides

The column was placed in a 2-mL microcentrifuge tube and centrifuged at ×1,000 g for 30 s to remove the storage buffer. Then, dissolve the peptide dry powder in 200 µL of binding/wash buffer and centrifuged at the same rate, and repeated once again. For phosphorylated peptide enrichment, 200 µL of the suspended peptide sample was first added to an equilibrated centrifuge column and the resin was mixed with the sample by tapping the bottom stopper and incubated for 30 min. The column was then placed in a microcentrifuge tube and centrifuged at ×1,000 g for 30 s before discarding the flow-through solution. The column was then washed with 200 µL of the binding/wash buffer, centrifuged at ×1,000 g for 30 s (repeated twice), and the flow-through solution was finally discarded. Then, 200 µL of the LC-MS-grade water was added to wash the column and centrifuged at ×1,000 g for 30 s. Finally, the column was placed in a new microcentrifuge tube, and 100 µL of the elution buffer was added to the column, centrifuged at ×1,000 g for 30 s (repeated once again). The eluate was then immediately dried in a high-speed vacuum concentrator to remove the elution buffer.

### 2.9 LC-MS/MS analysis

The peptides were dissolved in buffer A (0.1% formic acid [FA]), separated using an EASY-nLC 1200 UPLC system (ThermoFisher), and then sampled onto a 25 cm C18 (RP-C18, ionopticks) analytical column at a flow rate of 300 nL/min for gradient elution. B buffer (80% ACN plus 0.1% FA) was set as follows: 3%–27% for 0–66 min; 27%–46% for 66–73 min; 46%–100% for 73–84 min; and 100% for 84–90 min. The peptides were separated by the UHPLC system and injected into an NSI ion source for ionization and then analyzed by the timsTOF Pro mass Spectrometer with three biological replicates.

### 2.10 Data processing

Raw MS data were disposed employing the MaxQuant version (v1.5.2.8) with FDR <0.01 at the protein, peptide, and modification levels. Enzyme specificity was set to trypsin and searches included cysteine carbamidomethylation as a fixed modification, protein N-acetylation, oxidation of methionine, and/or phosphorylation of Ser, Thr, Tyr residues as variable modifications. A maximum of two missed cleavages were allowed during protease digestion, and the peptide was required to be fully trypsinized.

### 2.11 Bioinformatics analysis

For the identified phosphorylation site corresponding proteins, each annotation information was extracted based on the Uniprot, KEGG, GO, KOG/COG, and other databases to mine the protein functions. GO/KEGG enrichment analysis of the proteins corresponding to the differentially phosphorylated sites was performed to characterise their function. *p* < 0.05 was considered to indicate statistical significance.

### 2.12 Protein–protein interaction network analysis

The STRING (v.10.5, https://string-db.org/) database is a database of predicted functional correlations among the proteins. A protein–protein interaction (PPI) network of differentially phosphorylated proteins was constructed by setting the criterion “confidence level to ≥0.7".

### 2.13 Statistical analysis

Figures were expressed by using the indicator of mean ± standard deviation, which also analyzed using the SPSS.25 software (IBM Corp.). Assessment of escape latency in MWM by repeated-measures analysis of variance (ANOVA). Between-group comparisons of MWM spatial probe tests, bodyweight, and serological indicators were conducted by one-way ANOVA and Fisher’s least significant difference test. Data that did not satisfy the normal distribution were compared using non-parametric tests, expressed as median and interquartile spacing [M (P25%, P75%)], and differences between groups were compared using Bonferroni. *p* < 0.05 was considered to indicate statistical significance.

## 3 Results

### 3.1 Changes in body weight, blood glucose, and blood lipids in four groups of mice

**FIGURE 1:**
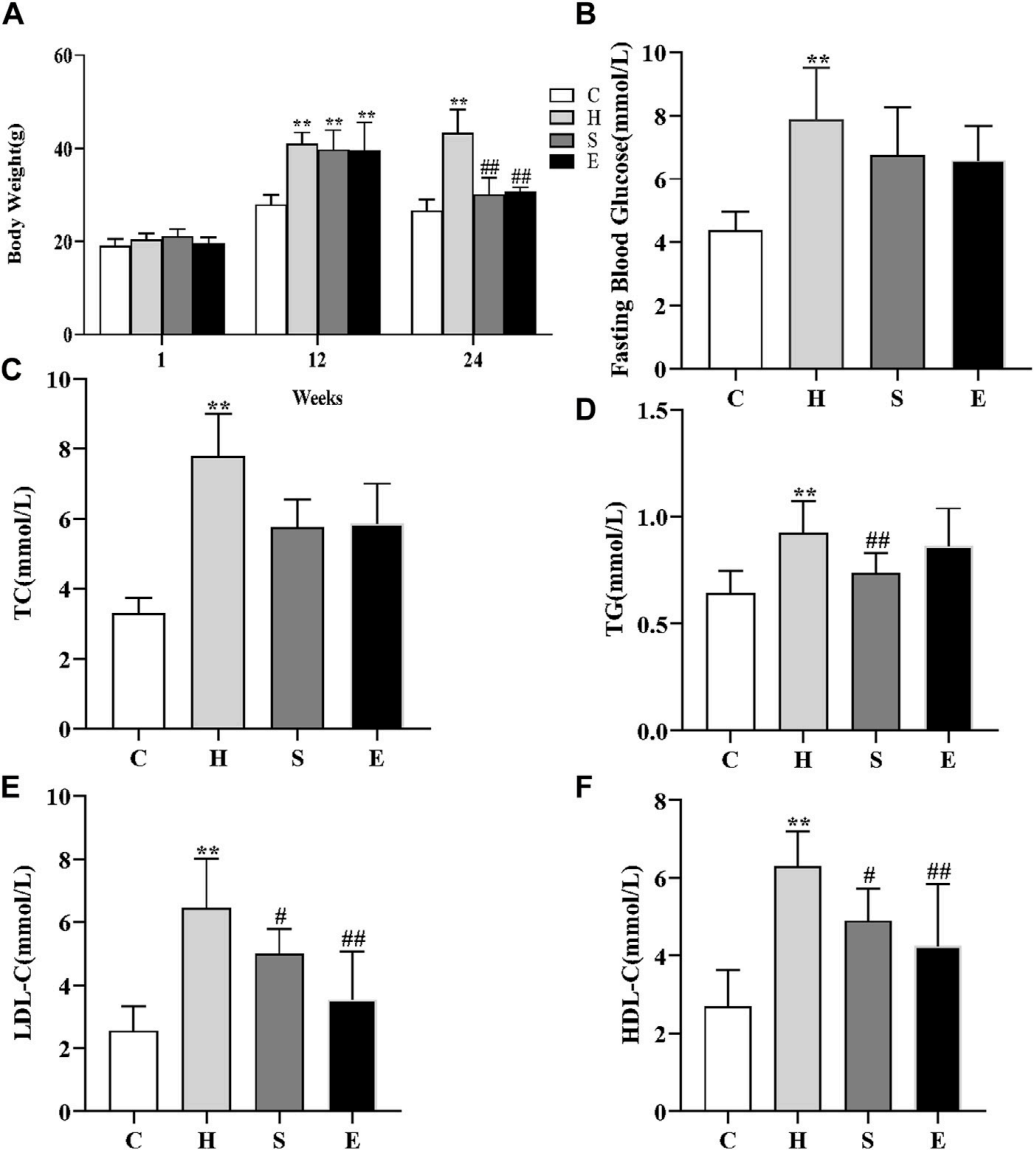
Effects of semaglutide and empagliflozin treatment on the metabolic parameters in HFD-induced obese mice. n = 8 per group. **(A)** High-fat diet increased the bodyweight of mice in group H, while the application of semaglutide and empagliflozin intervention significantly decreased the bodyweight of mice in groups S and E. **(B)** Comparison of the fasting blood glucose levels of mice in different groups; comparison of TC **(C)**, TG **(D)**, LDL-C **(E)**, HDL-C **(F)** in different groups. Values are expressed as mean ± SD or median and interquartile spacing [M (P25%, P75%)]. *p < 0.05 and **p < 0.01 vs. C;^#^ *p* < 0.05 and ^##^ *p* < 0.01 vs. H.

At first, there was no significant difference in bodyweight between the four groups of mice. After 12 weeks of high-fat feeding, the bodyweight of mice in group H increased significantly compared to group C, increasing more than 20%, and there was a significant difference between the two groups (27.93 ± 2.16 vs. 40.96 ± 2.49, *p* < 0.001), as shown in Figure 1A, indicating that the mouse obesity model had been successfully established. The bodyweight of mice decreased significantly with the combination of semaglutide and empagliflozin, while the bodyweight of group S decreased more significantly (43.31 ± 5.03 vs. 30.06 ± 3.59, *p* < 0.001; 43.31 ± 5.03 vs. 30.68 ± 0.98, *p* < 0.001). When compared to group C, fasting blood glucose, TC, TG, LDL-C, and HDL-C levels were all increased in group H [3.32 (2.98, 3.72) vs. 7.77 (6.51, 9.07), *p* < 0.001; 4.36 ± 0.59 vs. 7.86 ± 1.65, *p* < 0.001; 0.64 ± 0.10 vs. 0.92 ± 0.15, *p* < 0.001; 2.55 ± 0.78 vs. 6.44 ± 1.58, *p* < 0.001; 2.71 ± 0.93 vs. 6.30 ± 0.90, *p* < 0.001]. However, after intervention with semaglutide and empagliflozin, TG, LDL-C, and HDL-C were significantly lower in groups S compared to group H (0.92 ± 0.15 vs. 0.74 ± 0.09, *p* = 0.009; 6.44 ± 1.58 vs. 4.99 ± 0.786, *p* = 0.026; 6.30 ± 0.90 vs. 4.90 ± 0.83, *p* = 0.017), and fasting blood glucose and TC decreased compared to group H, but there was no statistical difference [7.86 ± 1.65 vs. 6.74 ± 1.52, *p* = 0.089; 7.77 (6.51, 9.07) vs. 5.89 (5.7, 6.24), *p* = 0.106]; LDL-C and HDL-C were significantly lower in group E compared to group H (6.44 ± 1.58 vs. 3.53 ± 1.53, *p* < 0.001; 6.30 ± 0.90 vs. 4.25 ± 1.59, *p* = 0.001), and fasting glucose, TC and TG decreased compared to group H, but there was no statistical difference [7.86 ± 1.65 vs. 6.59 ± 1.08, *p* = 0.056; 7.77 (6.51, 9.07) vs. 6.26 (4.55, 6.90), *p* = 0.445; 0.92 ± 0.15 vs. 0.86 ± 0.18, *p* = 0.366].

### 3.2 Semaglutide and empagliflozin reduce serum inflammatory markers in HFD-induced obese mice

**FIGURE 2:**
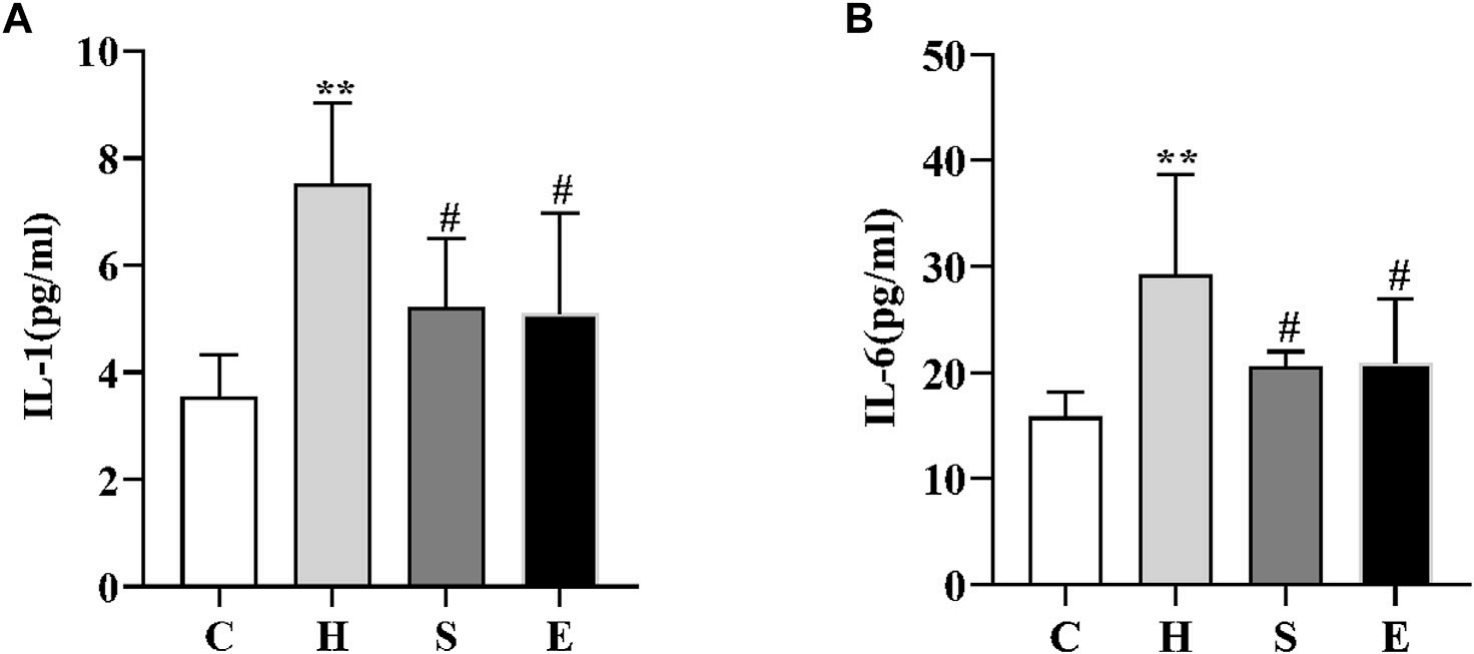
Comparison of IL-1 **(A)** and IL-6 **(B)** in mice in groups C, H, S, and E n = 5 per group. Values are expressed as mean ± SD. *p < 0.05 and **p < 0.01 vs. C; ^#^ *p* < 0.05 and ^##^ *p* < 0.01 vs. H.

We examined the changes in serum levels of IL-1 and IL-6. As shown in Figure 2, serum levels of IL-1 and IL-6 were significantly higher in group H compared to group C (3.55 ± 0.79 vs. 7.51 ± 1.51, *p* < 0.001; 15.80 ± 2.38 vs. 29.27 ± 9.47, *p* = 0.002); however, intervention with semaglutide and empagliflozin significantly reduced serum IL-1 and IL-6 levels in groups S and E, there was a statistically significant difference (7.51 ± 1.51 vs. 5.22 ± 1.27, *p* < 0.05; 29.27 ± 9.47 vs. 20.51 ± 1.47, *p* < 0.05; 7.51 ± 1.51 vs. 5.12 ± 1.86, *p* < 0.05; 29.27 ± 9.47 vs. 20.93 ± 6.03, *p* < 0.05).

### 3.3 Semaglutide and empagliflozin improve impairment of spatial learning and memory in HFD-induced obese mice

**FIGURE 3:**
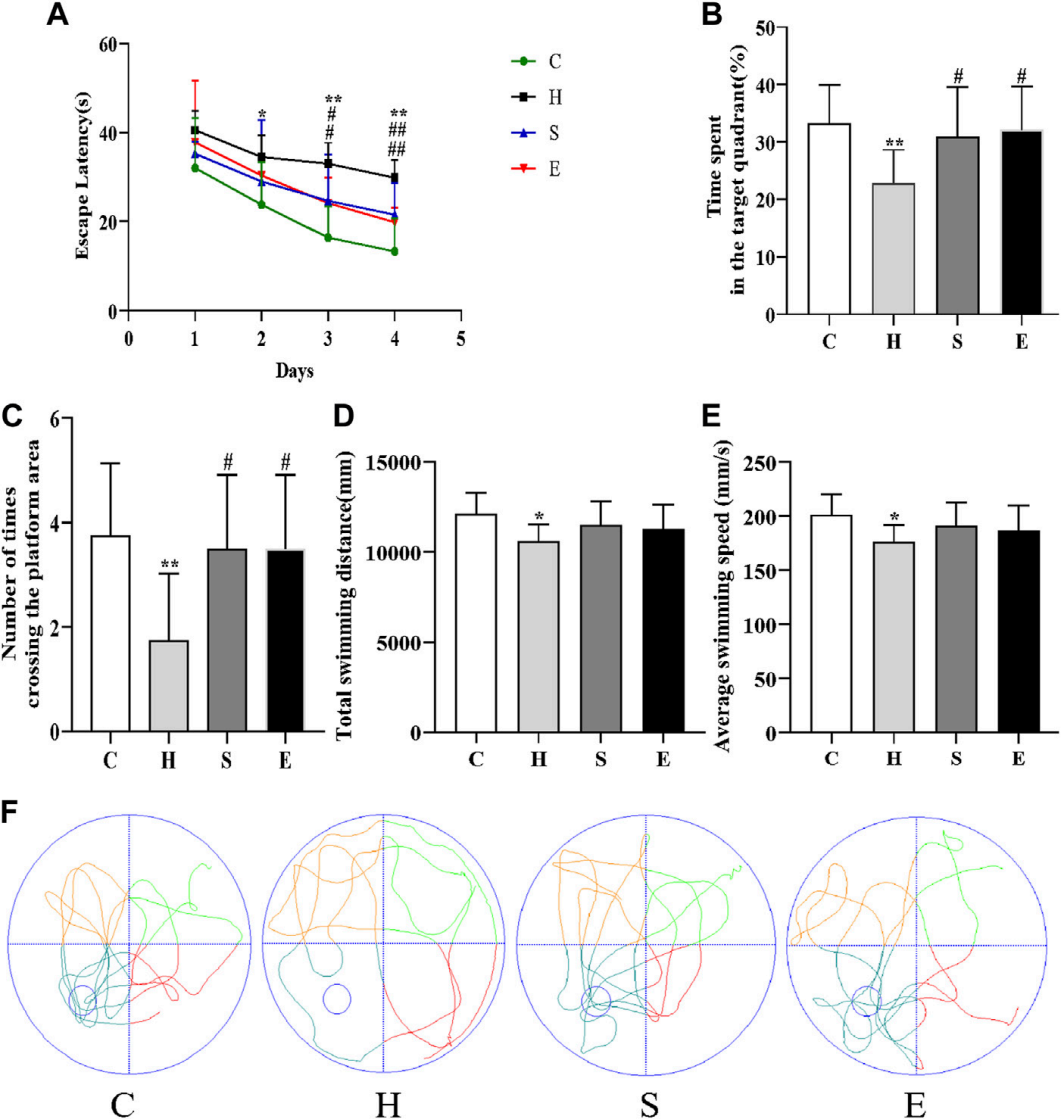
Semaglutide and empagliflozin alleviate learning memory deficits (Morris water maze, n = 8 per group). **(A)** Escape latency of mice from day 1 to day 4. **(B)** Time spent by mice in the target quadrant during the test. **(C)** Frequency of mice crossing the platform within 1 min during the test. **(D)** Total swimming distance of mice during the test. **(E)** Mean swimming speed of mice during the test. **(F)** Swimming path of mice during the test. Values are expressed as mean ± SD. *p < 0.05 and **p < 0.01 vs. C;^#^ *p* < 0.05 and ^##^ *p* < 0.01 vs. H.

As shown in Figure 3, the escape latency of mice in group H was longer than that of mice in group C on days 2–4 of the training phase (day 2: *p* < 0.05; day 3: *p* < 0.01; day 4: *p* < 0.01), indicating that high-fat diet-induced obesity leads to reduced spatial learning ability in mice. In contrast, the latency of mice in groups S and E was shorter than that of mice in group H (S vs. H: day 3: *p* < 0.05, day 4: *p* < 0.01; E vs. H: day 3: *p* < 0.05, day 4: *p* < 0.01), suggesting that semaglutide or empagliflozin therapy alleviated learning deficits. On the fifth day of the spatial probe test, HFD mice showed poor mobility, leading to a significant decrease in the total swimming distance and average swimming speed during the test period compared to group C mice (all *p* < 0.05), and a significant decrease in the time spent in the target quadrant and the number of platform crossings in group H (all *p* < 0.01). In contrast, groups S and E stayed in the target quadrant longer and had a higher frequency of platform crossing (all *p* < 0.05), but there was no statistical difference in total swimming distance and average swimming speed compared to group H mice (all *p* > 0.05). This is further supported by the swimming track (Figure 3F). In conclusion, both semaglutide and empagliflozin partially restored the deficit in spatial learning and memory.

### 3.4 Phosphorylation proteomics data overview

**FIGURE 4:**
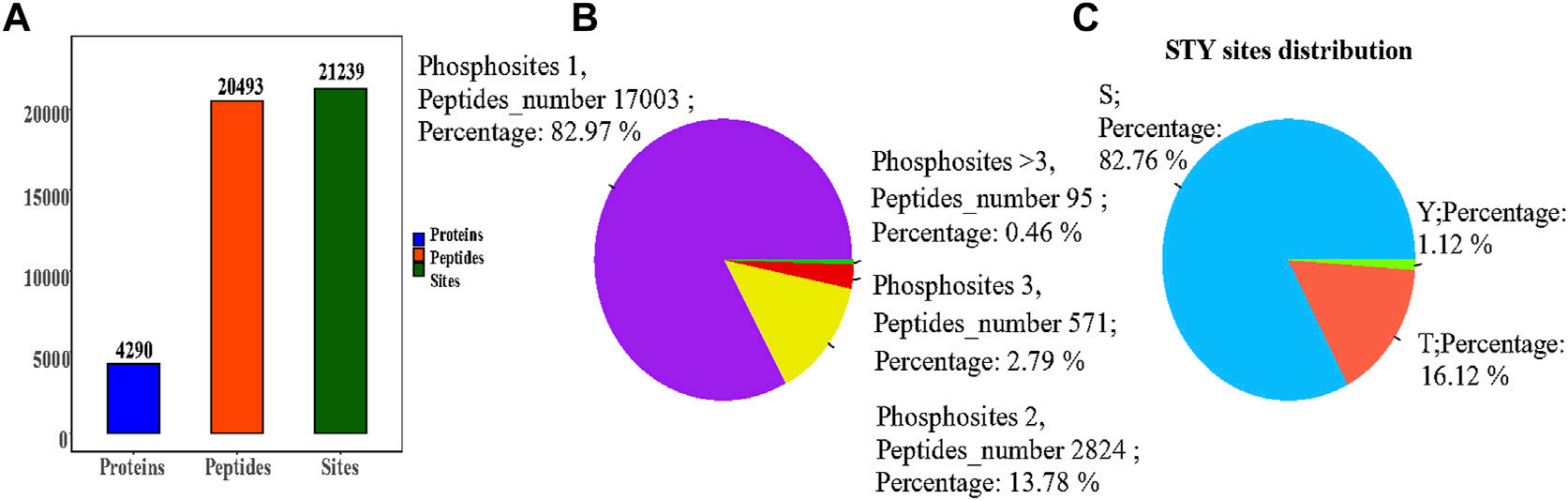
**(A)** Statistical information on the identification of the modifications; **(B)** Phosphosites-phosphorylation sites distribution graph, each sector in the pie chart indicates the number of phosphosites (Peptides number) for Phosphosites = 1, = 2, = 3, >3, respectively, and Percent indicates the percentage of peptides corresponding to each phosphosite case out of the total phosphosites; **(C)** Percentage of phosphorylation sites, with sectors indicating the percentage of S (serine), T (threonine), and Y (tyrosine) modifications in the total number of phosphorylation sites, respectively.

We applied the phosphorylation-based 4D-LFQ technique to identify and quantify phosphopeptides and phosphoproteins, identifying a total of 21,239 phosphorylation sites on 20,493 peptides and 4,290 phosphorylated proteins (Figure 4A)**.** Of the 20,493 detected phosphopeptides, 17,003 were uniquely phosphorylated, 2,824 were doubly phosphorylated, 571 were triple phosphorylated, and 95 had more than triple phosphorylated peptides (Figure 4B). In contrast, 17,577 of the 21,239 detected phosphorylation sites were located on serine residues, 3,424 on threonine residues, and 238 on tyrosine residues, accounting for 82.76, 16.12%, and 1.12%, respectively (Figure 4C).

Furthermore, sites with expression values ≥ 50% in any group of samples were retained based on the table of qualitative phosphorylation sites obtained from the screening. Loci with missing values ≤ 50% were filled with the mean values of the same group of samples, screened using Localization prob≥0.75 and Delta score≥8, and a total of 11,681 phosphorylation loci with high confidence were found using Median Normalization and log2 log transformation.

### 3.5 Identification of differential phosphorylation sites

**FIGURE 5:**
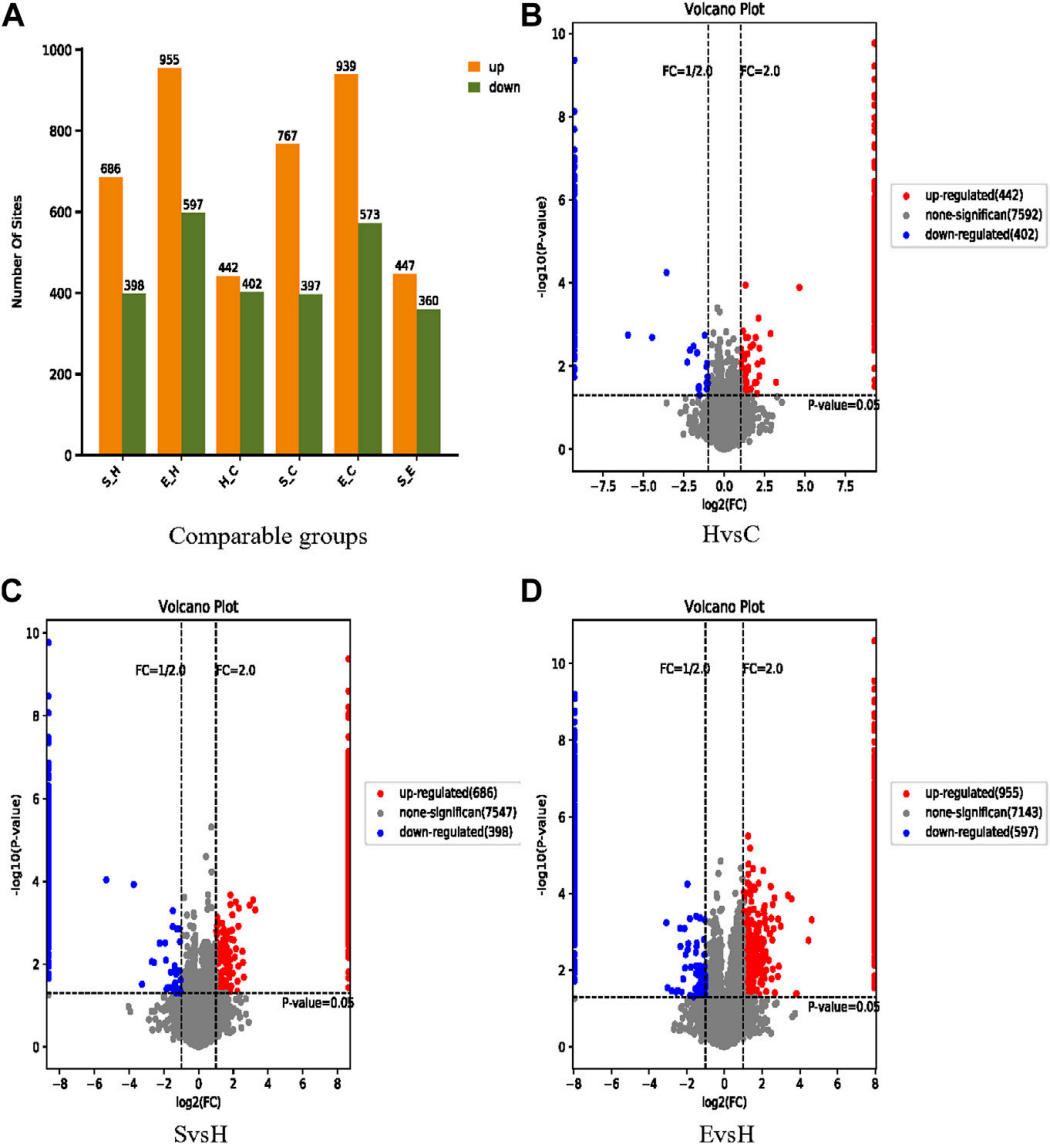
Information on the identification of differential phosphorylation sites. **(A)** Histograms of the distribution of the number of differentially phosphorylated sites in the different comparison groups; **(B–D)** volcano plots of differentially expressed proteins. The horizontal coordinate is log2 (FC), with values farther from 0 point indicating greater differences. The vertical coordinate is −log10 (*p*-value), with values farther from point 0 indicating greater variation. The blue points in the graph indicate down regulated differentially expressed proteins, the red points indicate up regulated differentially expressed proteins, and the grey points indicate non-significantly differentially expressed proteins.

Based on confidence loci, two criteria were selected to compute the difference between groups. The Fold change (FC) method was used to assess the fold change in expression levels of a locus across groups; the *p*-value derived by the *t*-test demonstrated the significance of the difference between groups. Figure 5A demonstrates a comparison of the expression of phosphorylated proteins in group S/H, group E/H, group H/C, group E/C and group S/E. 844 differentially significant phosphorylated loci were identified in the H/C group. There were 1,084 differentially significant phosphorylation sites identified in the S/H group, with 686 being upregulated and 398 being downregulated. In the E/H group, 1,552 differentially significant phosphorylation sites were identified, with 955 being upregulated and 597 being downregulated. We created volcano diagrams to depict the differentially expressed proteins more graphically, as shown in Figures 5B–D.

### 3.6 GO enrichment analysis of proteins corresponding to differentially phosphorylated sites

**FIGURE 6:**
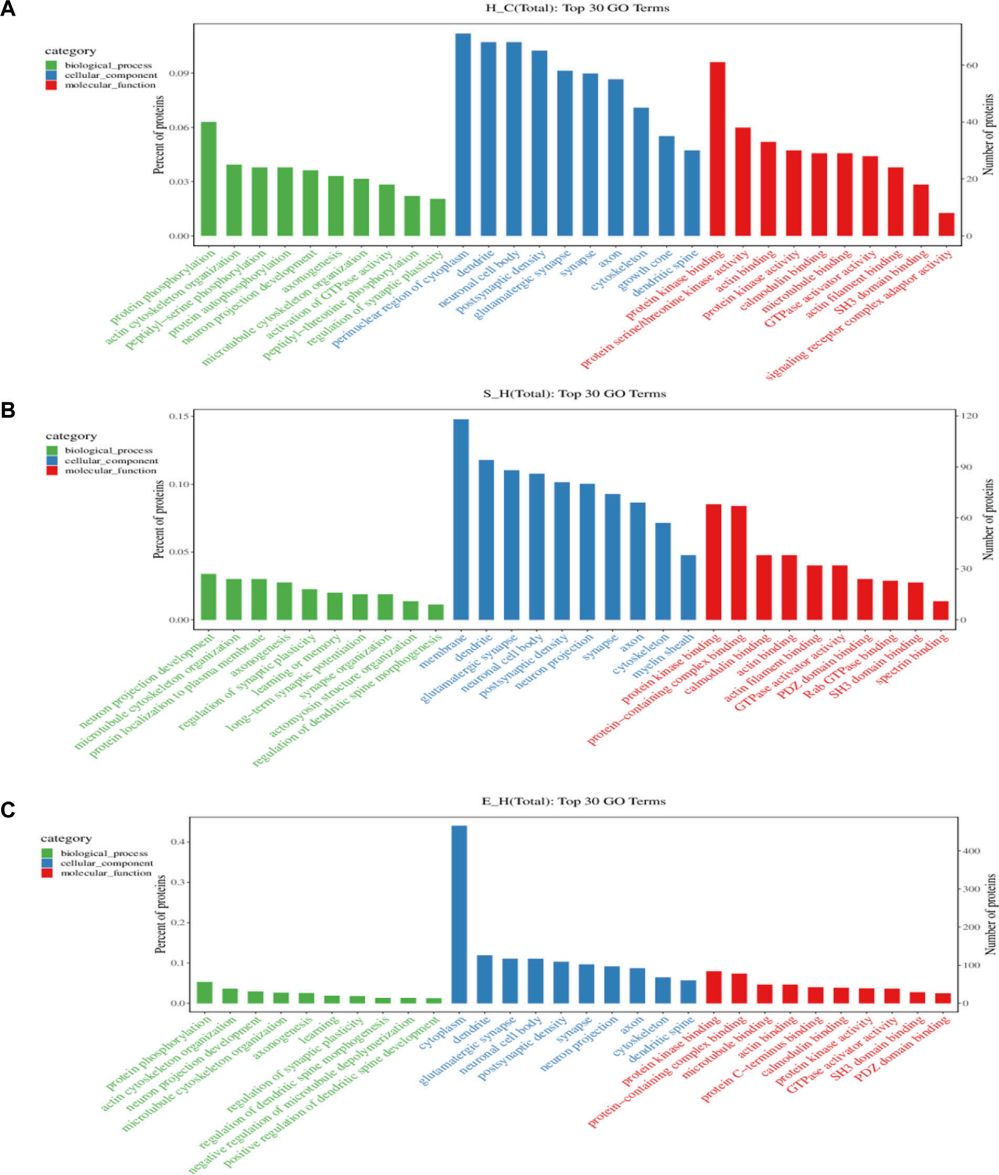
GO analysis of proteins corresponding to differentially phosphorylated sites. **(A–C)** functional classification of CC, MF, and BP in the H/C, S/H, and E/H groups. The horizontal coordinates of the graphs are the names of GO entries, and the vertical coordinates are the number of proteins corresponding to the entries and their percentages.

To gain more insight into the biological significance of differentially phosphorylated proteins, GO and KEGG enrichment analyses were performed, and the GO and KEGG enrichment results were clustered. As shown in Figure 6, the cellular components of differentially phosphorylated proteins in the H/C, S/H, and E/H groups were essentially the same in the CC analysis, with neuronal cell bodies being the most involved, followed by postsynaptic density, glutamatergic synapses, synaptic cell components, axons, and cytoskeleton. The three groups of differentially phosphorylated proteins in MF analysis mostly had molecular functions such as protein kinase binding, actin-binding, calmodulin-binding, GTPase activator activity, and SH3 structural domain binding. Further analysis showed that the three differentially phosphorylated proteins were primarily involved in neural projection development, synaptic plasticity control, axonogenesis, and microtubule cytoskeleton organization.

### 3.7 Subcellular structural localization and classification of proteins corresponding to differentially phosphorylated sites

**FIGURE 7:**
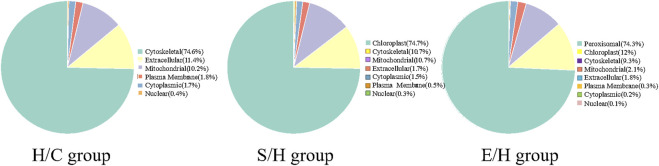
Subcellular localization map of differentially expressed phosphorylated proteins. The H/C and S/H group differentially expressed phosphorylated proteins were mainly distributed in the cell-matrix, while the E/H group differentially expressed phosphorylated proteins were mainly distributed in the peroxisome.

The differentially expressed phosphorylated proteins were further localized and classified subcellularly, and the results revealed that the differentially phosphorylated proteins in the three comparison groups of H/C, S/H, and E/H were mainly distributed in subcellular structures such as cytoskeletal, mitochondrial, cytoplasmic, and peroxisomal (Figure 7).

### 3.8 Analysis of the KEGG pathway of proteins corresponding to differentially phosphorylated sites

**FIGURE 8:**
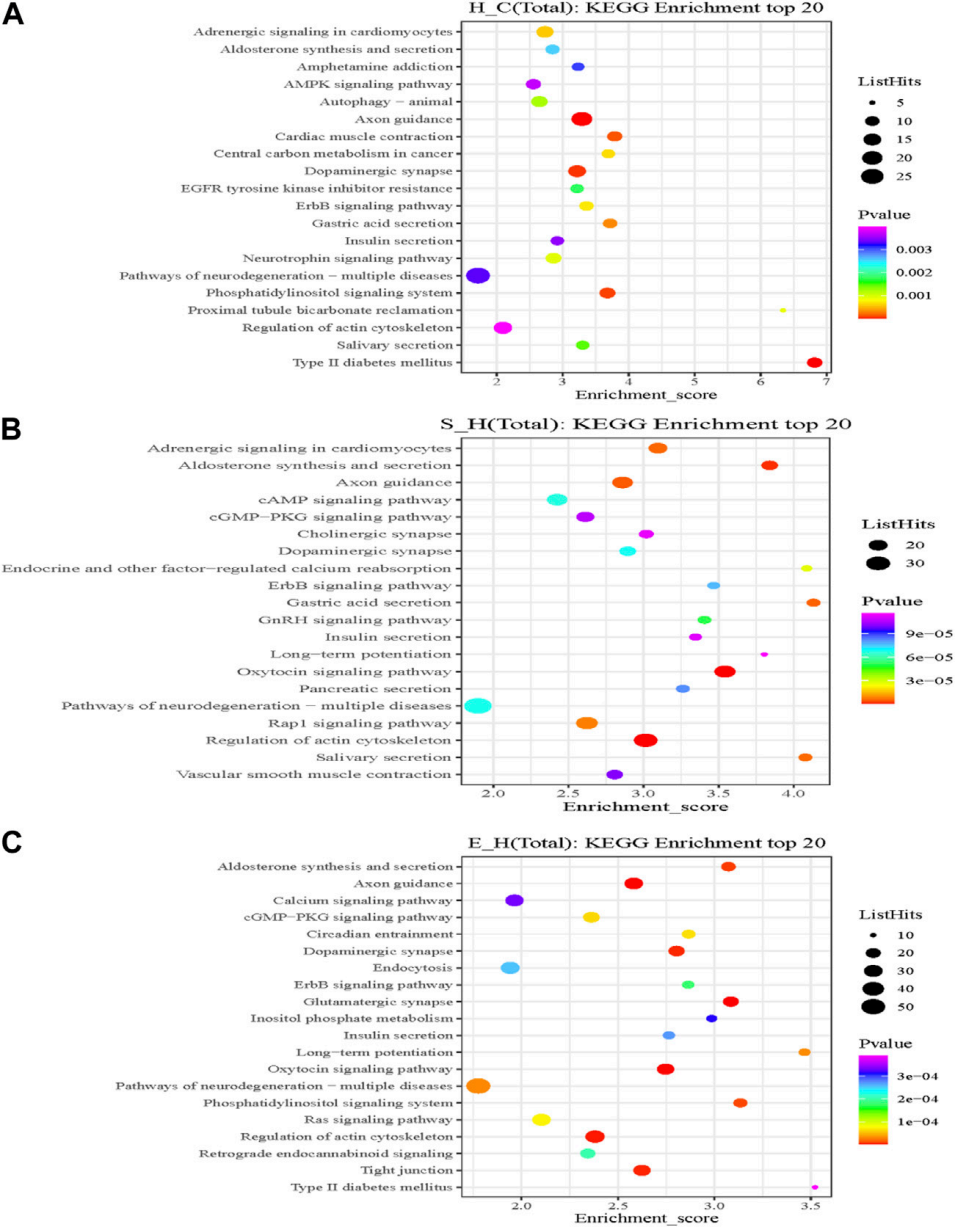
A rich bubble map of the Kyoto Gene Encyclopedia and the genomic pathway analysis. Differentially expressed proteins in the **(A)** H/C, **(B)** S/H, and **(C)** E/H groups. The *X*-axis Enrichment Score in the graph indicates the enrichment score and the *Y*-axis gives the top 20 pathway information. The larger the bubble, the more is the number of entries containing the number of proteins corresponding to the differential sites; the bubble color changed from red to green to blue to purple; and the smaller the enrichment *p*-value, the greater the significance.

**FIGURE 9:**
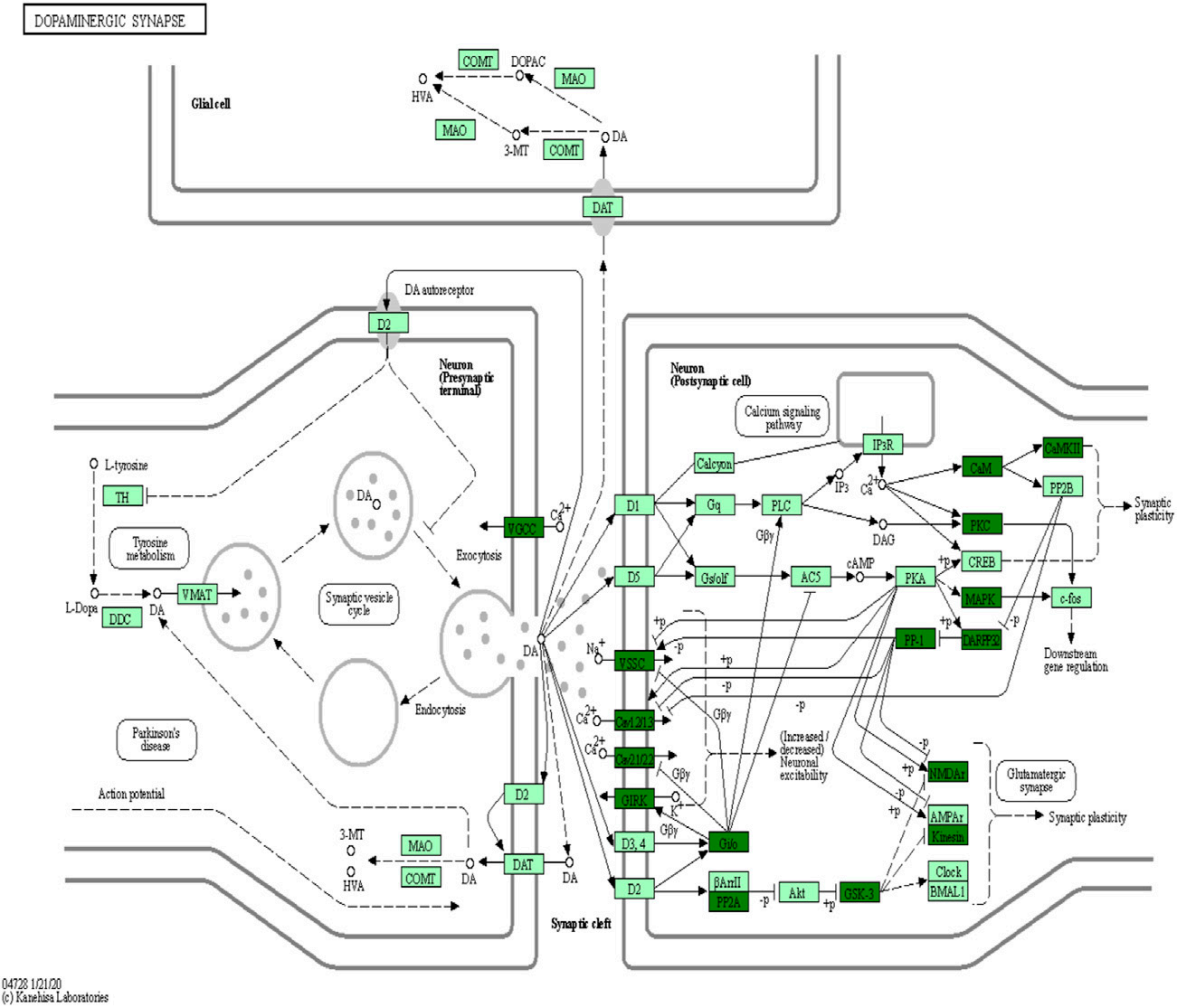
Dopaminergic synapse networks.

KEGG is the most widely used public database for studying protein metabolic pathways in cells. Using the KEGG database, the proteins that were differentially phosphorylated in the H/C group were found to be involved in dopaminergic synapses, axon guidance, myocardial contraction, central carbon metabolism in cancer, neurotrophic factor signaling pathways, and pathways of neurodegeneration—Metabolic pathways in multiple diseases (Figure 8A). The S/H group, on the other hand, showed differential phosphorylated proteins mainly focused on dopaminergic synapses, MAPK signaling pathway, cholinergic synapses, oxytocin signaling pathway, regulation of the actin cytoskeleton, and axon guidance (Figure 8B). In contrast, the differentially phosphorylated proteins in the E/H group were mainly focused on dopaminergic synapses, neurodegenerative pathways-multiple diseases, regulation of the actin cytoskeleton, axon guidance, myocardial contraction, ErbB signaling pathway, and insulin secretory metabolic pathway (Figure 8C). Furthermore, we discovered that the differentially phosphorylated proteins in the three comparison groups act together in dopaminergic synapse and axon guidance signaling pathway processes by combining the aforementioned findings. This is consistent with previous GO analyses, which found that proteins were concentrated in neuronal projection development, regulation of synaptic plasticity, and axonogenesis findings. Figure 9 depicts the dopaminergic synapse pathway.

### 3.9 Analysis of protein interaction networks corresponding to differentially phosphorylated loci

**FIGURE 10:**
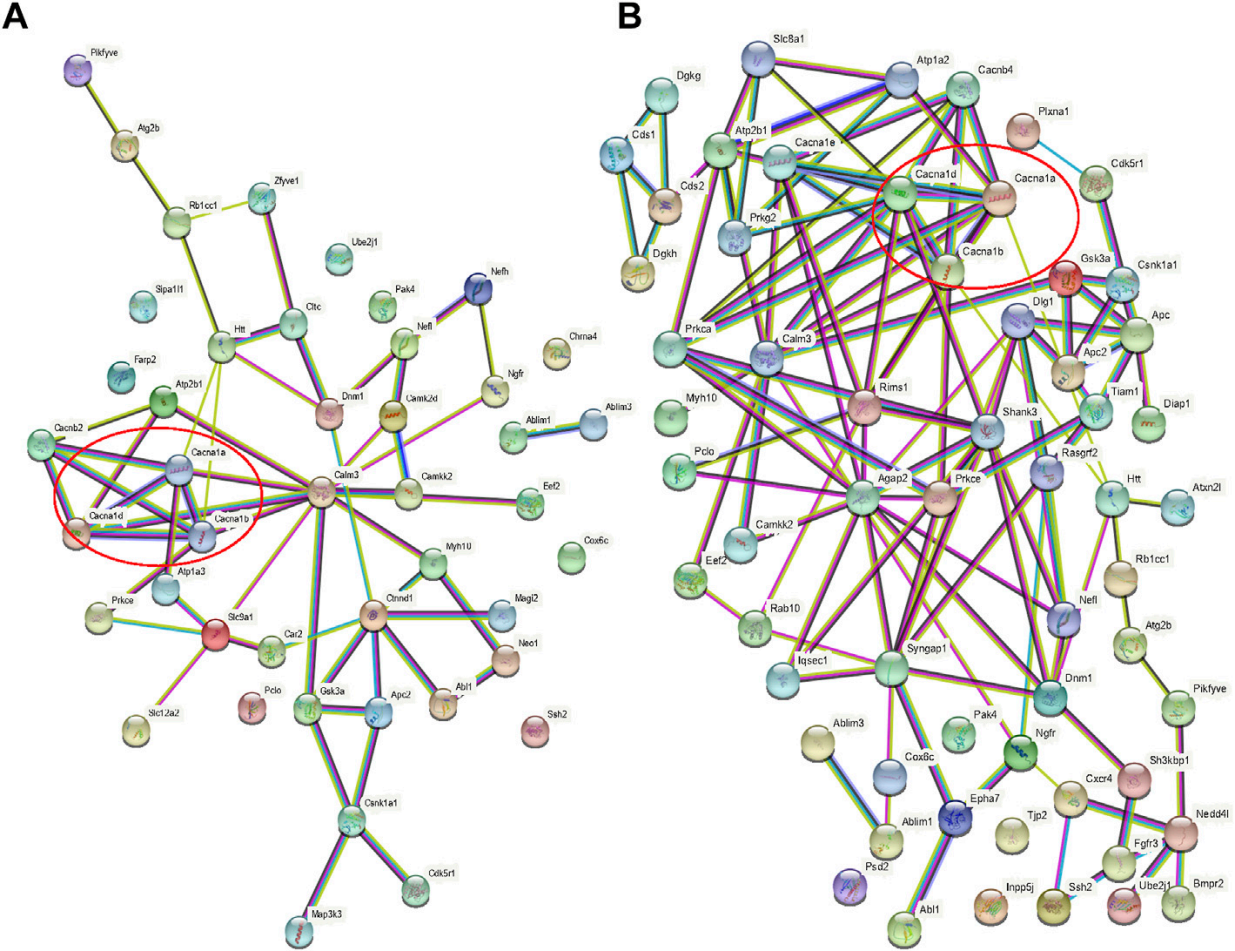
Protein interaction networks. **(A)** Interaction network of proteins with opposite direction of phosphorylation sites in the H/C and S/H groups and involved in the first 20 KEGG pathways; **(B)** Interaction network of proteins with an opposite direction of phosphorylation sites in the H/C and E/H groups and involved in the first 20 KEGG pathways. Red circles represent the interaction network among CACNA1D, CACNA1A, and CACNA1B.

When the results of differentially phosphorylated proteins from groups H and C, S and H, and E and H were combined, 44 proteins were found to be upregulated (or downregulated) at phosphorylation sites in group H and downregulated (or upregulated) at phosphorylation sites in group S and involved in the first 20 KEGG pathways (Table 1), and 60 proteins were found to be upregulated (or downregulated) at phosphorylation sites in group H and downregulated (or upregulated) at phosphorylation sites in group E and involved in the first 20 KEGG pathways (Table 2). The interactions of the proteins corresponding to the differential sites were obtained using the STRING database, it was discovered that voltage-dependent L-type calcium channel subunit alpha-1D (CACNA1D), voltage-dependent P/Q-type calcium channel subunit alpha-1A (CACNA1A), and voltage-dependent N-type calcium channel subunit alpha-1B (CACNA1B) in the different comparison groups were highly functionally related and operated together in the dopaminergic synapse pathway (Figures 9, 10).

**TABLE 1:** Proteins corresponding to differential phosphorylation sites reversed by semaglutide treatment.

Gene name	Description	H/C	S/H
CACNA1D	Voltage-dependent L-type calcium channel subunit alpha-1D	Down	Up
CACNA1A	Voltage-dependent P/Q-type calcium channel subunit alpha-1A	Down	Up
CACNA1B	Voltage-dependent N-type calcium channel subunit alpha-1B	Down	Up
Calm3	Calmodulin-3	Down	Up
Atp2b1	Plasma membrane calcium-transporting ATPase 1	Down	Up
Slc9a1	Sodium/hydrogen exchanger 1	Down	Up
Atp1a3	Sodium/potassium-transporting ATPase subunit alpha-3	Down	Up
Pak4	Serine/threonine-protein kinase PAK 4	Down	Up
Slc12a2	Solute carrier family 12 member 2	Down	Up
Myh10	Myosin-10	Down	Up
Abl1	Tyrosine-protein kinase ABL1	Down	Up
Prkce	Protein kinase C epsilon type	Down	Up
Ube2j1	Ubiquitin-conjugating enzyme E2 J1	Down	Up
Pikfyve	1-phosphatidylinositol 3-phosphate 5-kinase	Down	Up
Pclo	Protein piccolo	Down	Up
Ngfr	Tumor necrosis factor receptor superfamily member 16	Down	Up
Neo1	Neogenin	Down	Up
Nefl	Neurofilament light polypeptide	Down	Up
Magi2	Membrane-associated guanylate kinase, WW and PDZ domain-containing protein 2	Down	Up
Eef2	Elongation factor 2	Down	Up
Csnk1a1	Casein kinase I isoform alpha	Down	Up
Cltc	Clathrin heavy chain 1	Down	Up
Cdk5r1	Cyclin-dependent kinase 5 activator 1	Down	Up
Atg2b	Autophagy-related protein 2 homolog B	Down	Up
Camk2d	Calcium/calmodulin-dependent protein kinase type II subunit delta	Up	Down
Ssh2	Protein phosphatase Slingshot homolog 2	Up	Down
Cacnb2	Voltage-dependent L-type calcium channel subunit beta-2	Up	Down
Ca2	Carbonic anhydrase 2	Up	Down
Zfyve1	Zinc finger FYVE domain-containing protein 16	Up	Down
Sipa1l1	Signal-induced proliferation-associated 1-like protein 1	Up	Down
Rb1cc1	RB1-inducible coiled-coil protein 1	Up	Down
Nefh	Neurofilament heavy polypeptide	Up	Down
Map3k3	Mitogen-activated protein kinase kinase kinase 3	Up	Down
Htt	Huntingtin	Up	Down
Farp2	FERM, ARHGEF and pleckstrin domain-containing protein 2	Up	Down
Dnm1	Dynamin-1	Up	Down
Ctnnd1	Catenin delta-1	Up	Down
Cox6c	Cytochrome c oxidase subunit 6C	Up	Down
Chrna4	Neuronal acetylcholine receptor subunit alpha-4	Up	Down
Camkk2	Calcium/calmodulin-dependent protein kinase kinase 2	Up	Down
Ablim3	Actin-binding LIM protein 3	Up/down	Down/up
Ablim1	Actin-binding LIM protein 1	Up/down	Down/up
Apc2	Adenomatous polyposis coli protein 2	Up/down	Down/up
Gsk3a	Glycogen synthase kinase-3 alpha	Up/down	Down/up

**TABLE 2:** Proteins corresponding to differential phosphorylation sites reversed after empagliflozin treatment.

Gene name	Description	H/C	E/H
CACNA1D	Voltage-dependent L-type calcium channel subunit alpha-1D	Down	Up
CACNA1A	Voltage-dependent P/Q-type calcium channel subunit alpha-1A	Down	Up
CACNA1B	Voltage-dependent N-type calcium channel subunit alpha-1B	Down	Up
Ube2j1	Ubiquitin-conjugating enzyme E2 J1	Down	Up
Tjp2	Tight junction protein ZO-2	Down	Up
Syngap1	Ras/Rap GTPase-activating protein SynGAP	Down	Up
Ssh2	Protein phosphatase Slingshot homolog 2	Down	Up
Sh3kbp1	SH3 domain-containing kinase-binding protein 1	Down	Up
Rab10	Ras-related protein Rab-10	Down	Up
Psd2	PH and SEC7 domain-containing protein 2	Down	Up
Prkce	Protein kinase C epsilon type	Down	Up
Prkca	Protein kinase C alpha type	Down	Up
Pikfyve	1-phosphatidylinositol 3-phosphate 5-kinase	Down	Up
Pak4	Serine/threonine-protein kinase PAK 4	Down	Up
Ngfr	Tumor necrosis factor receptor superfamily member 16	Down	Up
Nefl	Neurofilament light polypeptide	Down	Up
Myh10	Myosin-10	Down	Up
Iqsec1	IQ motif and SEC7 domain-containing protein 1	Down	Up
Eef2	Elongation factor 2	Down	Up
Dlg1	Disks large homolog 1	Down	Up
Dgkh	Diacylglycerol kinase eta	Down	Up
Dgkg	Diacylglycerol kinase gamma	Down	Up
Csnk1a1	Casein kinase I isoform alpha	Down	Up
Cds2	Phosphatidate cytidylyltransferase 2	Down	Up
Cdk5r1	Cyclin-dependent kinase 5 activator 1	Down	Up
Calm3	Calmodulin-3	Down	Up
Cacnb4	Voltage-dependent L-type calcium channel subunit beta-4	Down	Up
Cacna1e	Voltage-dependent R-type calcium channel subunit alpha-1E	Down	Up
Atg2b	Autophagy-related protein 2 homolog B	Down	Up
Apc2	Adenomatous polyposis coli protein 2	Down	Up
Apc	Adenomatous polyposis coli protein	Down	Up
Ablim3	Actin-binding LIM protein 3	Down	Up
Ablim1	Actin-binding LIM protein 1	Down	Up
Abl1	Tyrosine-protein kinase ABL1	Down	Up
Prkg2	cGMP-dependent protein kinase 2	Up	Down
Pclo	Protein piccolo	Up	Down
Tiam1	T-lymphoma invasion and metastasis-inducing protein 1	Up	Down
Slc8a1	Sodium/calcium exchanger 1	Up	Down
Shank3	SH3 and multiple ankyrin repeat domains protein 3	Up	Down
Rims1	Regulating synaptic membrane exocytosis protein 1	Up	Down
Rb1cc1	RB1-inducible coiled-coil protein 1	Up	Down
Rasgrf2	Ras-specific guanine nucleotide-releasing factor 2	Up	Down
Plxna1	Plexin-A1	Up	Down
Nedd4l	E3 ubiquitin-protein ligase NEDD4-like	Up	Down
Inpp5j	Phosphatidylinositol 4,5-bisphosphate 5-phosphatase A	Up	Down
Htt	Huntingtin	Up	Down
Gsk3a	Glycogen synthase kinase-3 alpha	Up	Down
Fgfr3	Fibroblast growth factor receptor 3	Up	Down
Epha7	Ephrin type-A receptor 7	Up	Down
Dnm1	Dynamin-1	Up	Down
Diaph1	Protein diaphanous homolog 1	Up	Down
Cxcr4	C-X-C chemokine receptor type 4	Up	Down
Cox6c	Cytochrome c oxidase subunit 6C	Up	Down
Cds1	Phosphatidate cytidylyltransferase 1	Up	Down
Camkk2	Calcium/calmodulin-dependent protein kinase kinase 2	Up	Down
Bmpr2	Bone morphogenetic protein receptor type-2	Up	Down
Atxn2l	Amphiphysin	Up	Down
Atp2b1	Plasma membrane calcium-transporting ATPase 1	Up	Down
Atp1a2	Sodium/potassium-transporting ATPase subunit alpha-2	Up	Down
Agap2	Arf-GAP with GTPase, ANK repeat and PH domain-containing protein 2	Up	Down

## 4 Discussion

Obesity is a major risk factor for cognitive impairment such as AD and vascular dementia (Kim and Kim, 2021; Zhuang et al., 2021). The results of this study showed that fasting glucose, TC, TG, LDL-C and HDL-C levels were increased in mice in group H compared with group C. However, fasting glucose, TC, TG, LDL-C and HDL-C were decreased in groups S and E compared with group H after the intervention of semaglutide and empagliflozin. In addition, the obesity has been found in studies to influence brain structure, resulting in reduced brain volume, neuronal loss, and altered functional brain activity, thereby increasing the incidence of cognitive impairment (Tanaka et al., 2020). However, the pathogenesis of the effects of obesity on the central nervous system has not been fully elucidated. GLP-1 receptor agonists were first utilized as second-line hypoglycemic agents, but further studies have shown that GLP-1 has anti-inflammatory and neuroprotective effects, as well as the ability to improve cognitive impairment (Koshal et al., 2018). Furthermore, empagliflozin showed anti-inflammatory and antioxidant properties as well as enhanced effects on the brain-derived neurotrophic factor (BDNF), which regulates neurotransmission and ensures neuronal growth, survival, and plasticity in a diabetic mouse model (Chang et al., 2020). However, no reports have been published on the effects of semaglutide and empagliflozin on obesity-related cognitive impairment. In this study, we used phosphorylation 4D-LFQ technology for the first time to compare the phosphorylated proteomic expression profiles of hippocampal tissues from healthy, obese, semaglutide and empagliflozin treated mice, and applied bioinformatics methods such as GO functional analysis and KEGG pathway enrichment analysis to annotate and categorize the phosphorylated peptides in detail, revealing the characteristics of these differentially phosphorylated proteins, and providing a basis for the final screening of proteins that play a role in the development of obesity-related cognitive impairment. The results also revealed the characteristics of these differentially phosphorylated proteins, enabling the screening of phosphorylated proteins that may play a key role in the development of obesity-related cognitive impairment.

The most important clinical manifestation of cognitive impairment is learning and memory impairment. The Morris water maze experiment was used in this study to assess the learning and memory capacity of mice in different treatment groups. The findings revealed that high-fat diet-induced obese mice had prolonged escape latencies, reduced percentage of swimming time in the target quadrant, and a reduced number of stage penetrations than normal controls, indicating that obese mice have impaired short-term working memory and long-duration spatial reference memory abilities. We found for the first time that these indices were partially restored in obese mice treated with semaglutide and empagliflozin. Group H mice also showed a reduction in swimming time, which may not be related to memory performance, but rather a result of poorer mobility.

The phosphorylation 4D-LFQ technique was used to identify 21,239 phosphorylation sites on 20,493 peptides and 4,290 phosphorylated proteins for the first time in this work. Serine, threonine, and tyrosine accounted for 82.76%, 16.12%, and 1.12% of the 21,239 phosphorylation sites found, respectively (Figure 4). The differentially phosphorylated proteins in the three comparison groups of H/C, S/H, and E/H were mainly distributed in subcellular structures such as cytoskeletal, mitochondrial, cytoplasmic, and peroxisomal. In addition, KEGG enrichment analysis was performed on the differentially phosphorylated proteins in the different comparison groups. According to the findings, CACNA1D, CACNA1A, and CACNA1B were highly correlated among the differentially phosphorylated proteins in both treatment groups and were jointly enriched in the dopaminergic synapse pathway, which is consistent with the finding that the proteins were concentrated in the regulation of neuronal projection development, synaptic plasticity in GO analysis. HFD significantly decrease the phosphorylation of three key proteins, CACNA1DSer^802^, CACNA1ASer^2200^, and CACNA1BSer^936^. Semaglutide significantly increase CACNA1DSer^869^, CACNA1ASer^53^, and CACNA1BSer ^1,951,915^ phosphorylation, while empagliflozin significantly increase CACNA1DSer^1719^, CACNA1ASer^53^ and CACNA1BSer ^1951,915,892^ phosphorylation (Table 3). The main functions of the dopaminergic synapse pathway are the synthesis and release of dopamine and the activation of receptor signaling pathways that affect synaptic plasticity and thus regulate learning and memory. These three key proteins, CACNA1D, CACNA1A, and CACNA1B, can affect presynaptic neurotransmitter release and synaptic plasticity by mediating calcium inward flow, and L-type calcium channels can maintain pacing activity and therefore dopamine production, so we hypothesized that a high-fat diet impairs cognitive function in the hippocampus of mice by mediating reduced phosphorylation of CACNA1D at Ser^802^, CACNA1A at Ser^2200^, and CACNA1B at Ser^936^ in the dopaminergic synapse pathway to impair cognitive function in the hippocampus of mice, whereas semaglutide and empagliflozin may improve cognitive function in the hippocampus of mice by activating this pathway. This is a fresh discovery in the realm of metabolic disease and cognitive impairment research.

**TABLE 3:** Expression of Cacna1d, Cacna1a and Cacna1b in the Dopaminergic synapse pathway.

Group name	Gene name	Regulated type	*p*-Value	Positions within proteins	Amino acid	KEGG pathway	Fisher’s exact test *p*-value
H/C	CACNA1D	Down	3.119E-05	802	S	Dopaminergic synapse	0.000058
S/H	CACNA1D	Up	1.507E-06	869	S	Dopaminergic synapse	0.000069
E/H	CACNA1D	Up	0.001828	1719	S	Dopaminergic synapse	0.000005
H/C	CACNA1A	Down	3.283E-05	2,200	S	Dopaminergic synapse	0.000058
S/H	CACNA1A	Up	4.527E-06	53	S	Dopaminergic synapse	0.000069
E/H	CACNA1A	Up	2.124E-05	53	S	Dopaminergic synapse	0.000005
H/C	CACNA1B	Down	1.637E-05	936	S	Dopaminergic synapse	0.000058
S/H	CACNA1B	Up	0.0015003	1951	S	Dopaminergic synapse	0.000069
S/H	CACNA1B	Up	0.0030467	915	S	Dopaminergic synapse	0.000069
E/H	CACNA1B	Up	0.001166	1951	S	Dopaminergic synapse	0.000005
E/H	CACNA1B	Up	0.0037351	915	S	Dopaminergic synapse	0.000005
E/H	CACNA1B	Up	0.0021289	892	S	Dopaminergic synapse	0.000005

Voltage-gated Ca^2+^ channels (Cav) are transmembrane protein complexes composed of multiple subunits, and the genes encoding the α1 subunit of various voltage-gated calcium channels differ (Gandini and Zamponi, 2022). The CACNA1D gene encodes for the α1 subunit of the L-type calcium channel Cav1.3, which is widely expressed in several brain regions, including the hippocampus (Williams et al., 1992). L-Type Calcium Channels (LTCCs) are essential for normal fear, anxiety, and depression-like behaviors, as well as hippocampal-dependent cognitive function (Striessnig et al., 2014; Lauffer et al., 2022). Several studies have been reported on the effects of Cav1.3 on hippocampus-related learning and memory functions, however, their role is still debatable. Previous reports suggest that L-type calcium channel antagonists improve spatial memory in rodents while impairing learning and memory abilities (Quartermain et al., 2001; Woodside et al., 2004). Recent studies have shown that Cav1.3-deficient mice exhibit subtle deficits in normal brain development, as evidenced by reduced numbers of dopamine-producing neurons in the substantia nigra (Ortner et al., 2017) and reduced volume and fewer neurons in the auditory brainstem (Hirtz et al., 2012) and dentate gyrus (Marschallinger et al., 2015). The latter is associated with reduced hippocampal neurogenesis, inhibited neuronal differentiation, and hippocampal-dependent cognitive dysfunction (Marschallinger et al., 2015). In addition, Cav1.3-deficient mice also exhibit malfunction and degeneration of afferent auditory nerve fibers and hair cells, reduced functional synapse formation, and decreased survival of newborn neurons leading to hippocampal learning and memory impairment (Kim et al., 2017). This study found that a high-fat diet decreased the phosphorylation of CACNA1D in Ser^802^, which may affect neuronal development, synaptic plasticity and cognitive function in mice, which is consistent with previous findings. However, therapy with semaglutide and empagliflozin reduced spatial learning and memory dysfunction, which may be associated with increased hippocampal synaptic plasticity, neurogenesis, and CACNA1D expression.

Hippocampal synaptic transmission and activity-dependent synaptic plasticity play key roles in the performance of learning and memory functions. The Cav family’s CACNA1A gene encodes the alpha one subunit of the P/Q-type calcium channel CaV2.1 channel (Starr et al., 1991), whereas the CACNA1B gene encodes the alpha one subunit of the N-type calcium channel CaV2.2 (Mori et al., 1991). They both contain four repetitive structural domains, each with six transmembrane segments, and they both mediate calcium inward flow following membrane depolarization (Dolphin and Lee, 2020). CACNA1A is predominantly expressed in neuronal tissues and is implicated in mediating transmitter release from synapses and nerve endings. Its upregulation has been shown to alter synaptic strength (Lubbert et al., 2019), contribute to short-term plasticity (Yan et al., 2014; Nanou et al., 2016a), and contribute to long-term plasticity (Nanou et al., 2016b). CACNA1B gene mutations can alter transmitter release, resulting in dysregulation of synaptic transmission and neuronal network dysfunction (Bunda et al., 2019). Studies have reported that Cav regulates presynaptic membrane cytosolic processes and that most synapses are synchronized for release *via* the cooperative activation of Cav2.1 and/or Cav2.2, and ion channel blockers using both can delay impulse transmission in dopaminergic neurons (Yamamoto and Kobayashi, 2018). Under normal conditions, glutamate release is regulated by the Cav2.1 and Cav2.2 calcium channels. Whereas Cav2.1 currents are completely absent in CACNA1A knockout mice, hippocampal synaptic glutamate transmitter release becomes solely dependent on Cav2.2 channels, and transmitter release is reduced, implying that the absence of Cav2.1-type channels is not fully compensated for by Cav2.2 channels (Ayata et al., 2000). Recent findings indicate that Cav2.1 channel deficiency due to loss-of-function mutations in human CACNA1A can cause a variety of cognitive impairments and epileptic manifestations, such as cerebellar ataxia (Izquierdo-Serra et al., 2020), intellectual disability (Damaj et al., 2015), and childhood-onset refractory epileptic encephalopathy (Le Roux et al., 2021). In addition, the C-terminal region of the Cav2.2 channel is known to be involved in the tethering of synaptic vesicles (Wong et al., 2013), therefore mutations may interfere with this process. Notably, semaglutide and empagliflozin may restore this process by promoting CACNA1A serine phosphorylation, hence protecting cognitive function.

Notably, the neuroprotective activity of GLP-1 receptor agonists does not appear to be exclusively associated with weight normalization. Previous studies have shown that GLP-1 receptors present in the brain are also involved in cognition, synaptic transmission of hippocampal neurons and apoptosis. Overexpression of this receptor is responsible for cognitive enhancement and neuroprotection, while deficiency increases the chance of seizures and neurodegeneration (Salameh et al., 2020). A prospective study showed that liraglutide improved cognitive decline in patients with type 2 diabetes, and this beneficial effect was independent of its hypoglycemic effect and weight loss (Li et al., 2021). Our current data further reinforce that the improving effect of GLP-1 receptor agonists on cognitive function may not be entirely dependent on metabolic effects. Furthermore, recent studies have highlighted that in addition to metabolic changes, SGLT2i exhibits important neuroprotective properties (Kullmann et al., 2022). (Mone et al., 2022a) argue that empagliflozin improves cognitive impairment in frail older adults with T2DM and heart failure with preserved ejection fraction. Further studies have shown that empagliflozin reduces frailty in diabetic and hypertensive patients, most likely by decreasing the mitochondrial generation of reactive oxygen species in endothelial cells (Mone et al., 2022b). Therefore semaglutide and empagliflozin may be expected to be used to improve cognitive dysfunction, but further clinical evaluation is needed.

There are still some limitations in this study. First, we did not observe the effect of the combination of semaglutide and empagliflozin on cognitive function due to financial, time and experimental conditions, we speculate that the combination may have better effect than single drug, but further studies are still needed to confirm; second, the key genes and key signaling pathways screened were not validated, and there may be interactions between gene expression, resulting in inaccurate results, and further cell-related basic experimental studies will be conducted later; finally, our study found that the changes in genes may be correlated, but the causal relationship still needs further confirmation.

## 5 Conclusion

In conclusion, we found for the first time that a high-fat diet decreased CACNA1D, CACNA1A, and CACNA1B protein serine phosphorylation, which may affect neuronal development, synaptic plasticity, and cognitive function in mice. Notably, semaglutide and empagliflozin increased the phosphorylation of these proteins.

## Data Availability

The original contributions presented in the study are included in the article/Supplementary Materials. Further inquiries can be directed to the corresponding author.
